# Complete Genome Sequence of *Bacillus methylotrophicus* Strain NKG-1, Isolated from the Changbai Mountains, China

**DOI:** 10.1128/genomeA.01454-17

**Published:** 2018-01-18

**Authors:** Binghua Liu, Beibei Ge, Noreen Azhar, Wenjun Zhao, Hailan Cui, Kecheng Zhang

**Affiliations:** a Institute of Plant Protection, Chinese Academy of Agricultural Sciences, Beijing, China; b Institute of Biotechnology and Genetic Engineering, The University of Agriculture, Peshawar, Pakistan

## Abstract

We report here the complete genome sequence of *Bacillus methylotrophicus* NKG-1, isolated from rare dormant volcanic soils on the Changbai Mountains in China. The 4.20-Mb genome contains 4,432 genes and has a G+C content of 47.06%.

## GENOME ANNOUNCEMENT

*Bacillus methylotrophicus* is a Gram-positive bacterium associated with plant roots and has beneficial effects on the growth of many plants (1). The indole-3-acetic acid content was found to be 2- and 3-fold higher in the shoots and roots of bacterized seedlings, respectively, than in the control group (2). This strain can be used as a biofertilizer to promote plant growth and as a biopesticide to target root pathogens, including Gram-negative bacteria and fungi (3, 4). *B. methylotrophicus* NKG-1 was isolated from rare dormant volcanic soils on the Changbai Mountains in China. This strain is an effective biocontrol agent against 13 different kinds of soilborne phytopathogenic fungi and can promote the growth of tomato plants under both greenhouse and field conditions (5). This strain has been preserved at the China General Microbiological Culture Collection Center (CGMCC12055) and can be cultured at 28°C in Luria-Bertani (LB) medium.

Genome sequencing was performed using a third-generation PacBio RSII instrument. The use of a single-molecule sequencing approach has many advantages, including a long reading sequence, which facilitates easier genome assembly. The SMRT Portal software was employed to assemble reads into preliminary results (6, 7), and reads were compared with the assembled sequence to determine the sequencing depth. Chromosome and plasmid sequences were distinguished by sequence length and whether the sequence is annular. The GeneMarkS software (http://topaz.gatech.edu/GeneMark/genemarks.cgi) was used to predict bacterial protein-coding genes. Gene functional annotation was performed based on BLASTp searches against nonredundant (NR), Kyoto Encyclopedia of Genes and Genomes (KEGG) (8), Protein Family (Pfam) (9), and Cluster of Orthologous Groups (COG) (10) databases.

The *Bacillus methylotrophicus* genome is 4,197,217 bp, with a G+C content of 47.06%. A single chromosome containing 4,432 genes covering 3,768,021 bp, with an average gene length of 850 bp, accounts for ~89.77% of the genome. In addition, the genome encodes 114 structural RNAs, including 9 5S rRNAs, 9 16S rRNAs, 9 23S rRNAs, and 87 tRNAs.

A previous study showed that NKG-1 fermentation broth inhibits the growth of *Botrytis cinerea* on tomato seedling leaves *in vitro* by 60%, resulting in significant increases in seedling fresh weight (27.4%), seedling length (12.5%), and root length (57.7%) compared to the controls (5). The genome of *B. methylotrophicus* will be compared with those of other *Bacillus* strains in future research to illuminate the mechanisms promoting plant growth and biocontrol activity against fungal diseases, as well as to identify the compounds involved.

### Accession number(s).

The complete genome sequence of *Bacillus methylotrophicus* NKG-1 has been deposited in GenBank under accession no. CP024203.
